# Multifactorial Pathogenic Processes of Retinal Ganglion Cell Degeneration in Glaucoma towards Multi-Target Strategies for Broader Treatment Effects

**DOI:** 10.3390/cells10061372

**Published:** 2021-06-02

**Authors:** Gülgün Tezel

**Affiliations:** Department of Ophthalmology, Vagelos College of Physicians and Surgeons, Columbia University, Edward S. Harkness Eye Institute, 635 W 165th St., Box 102, New York, NY 10032, USA; gt2320@cumc.columbia.edu; Tel.: +1-212-342-3841

**Keywords:** glia, glaucoma, immunomodulation, neurodegeneration, neuroinflammation, neuroprotection, retinal ganglion cell

## Abstract

Glaucoma is a chronic neurodegenerative disease characterized by apoptosis of retinal ganglion cell (RGC) somas, degeneration of axons, and loss of synapses at dendrites and axon terminals. Glaucomatous neurodegeneration encompasses multiple triggers, multiple cell types, and multiple molecular pathways through the etiological paths with biomechanical, vascular, metabolic, oxidative, and inflammatory components. As much as intrinsic responses of RGCs themselves, divergent responses and intricate interactions of the surrounding glia also play decisive roles for the cell fate. Seen from a broad perspective, multitarget treatment strategies have a compelling pathophysiological basis to more efficiently manipulate multiple pathogenic processes at multiple injury sites in such a multifactorial neurodegenerative disease. Despite distinct molecular programs for somatic and axonal degeneration, mitochondrial dysfunction and glia-driven neuroinflammation present interdependent processes with widespread impacts in the glaucomatous retina and optic nerve. Since dysfunctional mitochondria stimulate inflammatory responses and proinflammatory mediators impair mitochondria, mitochondrial restoration may be immunomodulatory, while anti-inflammatory treatments protect mitochondria. Manipulation of these converging routes may thus allow a unified treatment strategy to protect RGC axons, somas, and synapses. This review presents an overview of recent research advancements with emphasis on potential treatment targets to achieve the best treatment efficacy to preserve visual function in glaucoma.

## 1. Introduction

**Figure 1:**
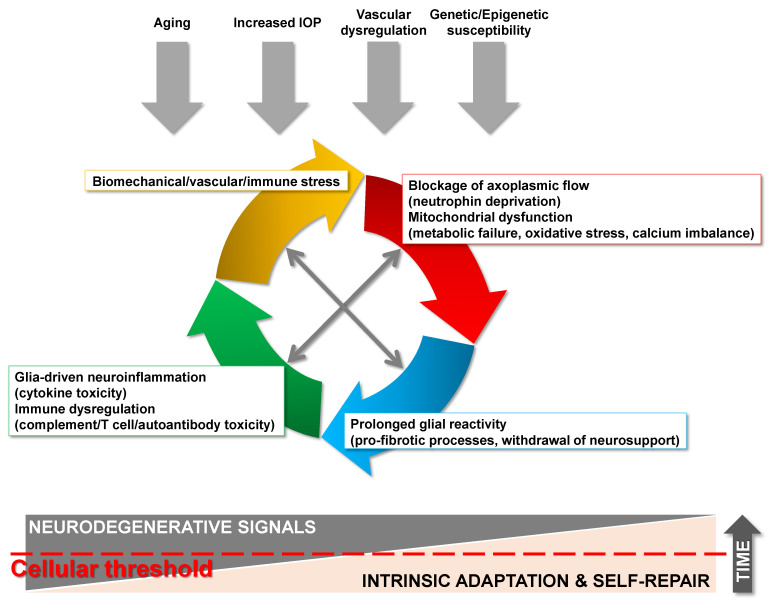
Glaucoma is a multifactorial neurodegenerative disease. The etiological framework of neurodegeneration in glaucoma encompasses multiple stressors, including increased intraocular pressure (IOP), aging, vascular dysfunction, and genetic/epigenetic factors. Interconnected pathogenic processes for glaucomatous neurodegeneration involves biomechanical, vascular, metabolic, oxidative, or inflammatory components. The asynchrony in RGC degeneration in glaucoma, along with the complexity of extrinsic triggers and intrinsic adaptive/reparative responses, suggest that a cellular stressor threshold determines the individual susceptibility of RGCs to injury in glaucoma.

Glaucoma is a chronic neurodegenerative disease leading to irreversible blindness. Progressive degeneration of retinal ganglion cells (RGCs) in glaucoma involves somatic apoptosis in the retina, axonal degeneration in the optic nerve, and synaptic loss at dendrites and axon terminals. The hallmark structural alterations detectable throughout the visual pathway are accompanied by alterations in functional connectivity of neural circuits, thereby leading to progressive loss of visual function in glaucoma patients (reviewed [1,2]). Growing information from experimental studies of glaucoma portrays an interconnected network of pathogenic processes for RGC degeneration. This network embraces biomechanical disruption of axonal transport, neurotrophin deprivation, mitochondrial dysfunction, metabolic failure, oxidative stress, calcium imbalance, vascular dysregulation, and neuroinflammation. Longstanding evidence depicts elevated intraocular pressure (IOP), at any magnitude, and aging as the most prevalent stressors for RGCs in glaucoma. Yet, it is increasingly recognized that beyond IOP and age-related stress, many other factors affect the vulnerability of RGCs to biomechanical, vascular, metabolic, oxidative, and inflammatory injury in glaucomatous eyes. Increased glaucoma susceptibility may have a genetic source in some families; however, identified genetic mutations have variable penetrance due to polygenic effects and complex interactions of genetic, epigenetic, and environmental risk factors [3,4]. By seeing the big picture, glaucomatous neurodegeneration encompasses an intricate interplay of multiple triggers, multiple cell types, and multiple molecular pathways. Relying on the asynchrony in degeneration of individual RGCs, the complexity of extrinsic triggers and intrinsic adaptive responses, and the contribution of both local and systemic factors, there appears to be a cellular threshold to withstand glaucomatous injury (Figure 1). Currently available treatments, which are limited to IOP lowering therapeutics, cannot prevent progressive vision loss from glaucoma. Consequently, this neurodegenerative disease remains a significant cause of blindness that currently affects 80 million people worldwide, and this number is estimated to reach over 100 million by the year 2040 [5,6]. Undoubtedly, only through an improved understanding of its multifactorial pathophysiology can effective treatment of this blinding disease be developed.

## 2. Complex Processes of Glaucomatous Neurodegeneration for Translation into Disease-Modifying Treatments to Preserve Neuron Survival and Visual Function

On the basis of human pathological studies and studies of animal models, the optic nerve head is a critical site of injury in glaucoma, where IOP-dependent or -independent insults can originate distal and proximal signals for axonal and somatic degeneration of RGCs. The health of RGC axons, somas, and synapses are ultimately dependent on one another, and the visual function can only be maintained with an intact neuronal connectivity. However, axonal self-destruction and somatic apoptosis are regulated by molecularly distinct pathways [7,8,9]. The self-autonomous destruct pathways for different RGC compartments include Wallerian degeneration of distal axons and Bax-dependent apoptosis of RGC somas. Evidently, glaucoma-related RGC soma death can be prevented by *Bax*^−/−^*,* whereas Bax deficiency is not sufficient to prevent axon degeneration [8,10]. On the other hand, the gain of function mutation of the Wallerian degeneration gene, *Wld^S^,* delays axon loss in ocular hypertensive rats [11] and mice [8] with no prominent effect on RGC somas. While the signals originating from an axonal insult promote RGC degeneration in the somatic compartment, anterograde signals from the RGC soma may also elicit axon degeneration. This is evident in Bax-deficient DBA/2J mice with hereditary glaucoma, in which axons distal to laminar region of the optic nerve head degenerate, but the proximal axon segments attached to rescued RGC somas stay intact [8,10]. Thus, proximal and distal axons also degenerate through distinct processes, as proximal axons appear to degenerate secondary to RGC apoptosis. Notwithstanding the evidence of independent molecular programs for somatic and axonal degeneration in glaucoma, there are also mechanistic overlaps and molecular connections [2].

Although initial axon injury is a major driver for RGC degeneration in glaucoma, whether the death of RGC somas merely results from an axonal insult, or whether direct injury to RGC somas may also initiate a degenerative cascade, is not fully understood. As a matter of fact, there are early retinal alterations that suggest the pathogenic processes arising from RGC somas and dendrites. Besides the signaling between RGC axons and somas, RGCs themselves can also sense and respond to IOP-related stress [12,13,14,15]. Early alterations of RGCs include silencing of the RGC-specific gene expression, which precedes neuron loss in animal models of glaucoma [16,17,18,19]. Pruning of RGC dendritic arbors and loss of RGC synapses with amacrine and bipolar neurons [20,21,22] are also among early alterations detected in the glaucomatous retina. The *Wld^S^* may prevent ocular hypertension-induced early changes in RGC dendrites [23]; however, whether this dendritic protection is related to preservation of axon integrity in these mice is difficult to interpret. In fact, a dendritic remodeling may even be detectable prior to dendrite atrophy and function loss [24,25]. These experimental observations are supported by pathological [26] and clinical studies of human glaucoma [27], which present localized abnormalities and remodeling of the retinal inner plexiform layer in correlation with early deficits in visual field function. Glaucoma models also suggest that besides RGCs, other retinal neurons, including amacrine cells, may be affected as well [28,29].

Early injury signals in glaucoma may reach brain synapses of RGCs, as manifested by reduced dendrite complexity and volume of the lateral geniculate nucleus in human glaucoma and animal models [30,31,32]. It is of interest to note that early disconnection of RGC synaptic terminals in the brain may also derive a dying back process progressing in a distal-to-proximal pattern from synaptic region towards cell body [8,33,34]. Multitude of injury sites warrants additional studies to better assess the time course and mechanistic interactions of anterograde and retrograde degeneration signals at different subcellular regions of RGCs in experimental glaucoma. Apparently, RGCs are specific victims of glaucoma; however, other cell types, mainly including the neighboring glia, are also decisive for the RGC fate. Neuroinflammation, relying on reactive glia, may consist of protective and reparative elements; however prolonged proinflammatory responses generate broadly damaging outcomes that can contribute to RGC soma death, axon degeneration, and synapse loss in glaucoma [35,36,37,38], as outlined later below.

### 2.1. Research Approaches to Glaucomatous Neurodegeneration

Using a wide variety of approaches to different viewpoints, glaucoma research aims for a unified and comprehensive understanding of this complex neurodegenerative disease. High throughput datasets accumulated from transcriptomics [39,40], proteomics [41], metabolomics [42], or lipidomics [43] analyses of animal models, or human donor tissues, provide hypothesis-generating frameworks. Following studies use pharmacologic or transgenic strategies to test new hypotheses and to identify molecular targets for new therapeutic interventions. Increasing availability of an array of powerful research tools for isolation, targeting, and analysis of specific cell types also enables cell type-specific analysis of glaucoma models, in vivo. Heterogeneous cellular composition of the retina and optic nerve, together with a divergent and dynamic nature of the responses of different cell types, make the cell type-specific analysis crucial for precise characterization of cellular responses during glaucomatous neurodegeneration. As much as for characterization of the sequence of molecular events in individual cell types, or in their subpopulations, cell type-specific information is essential for a thorough understanding of neuron-glia and glia-glia interactions. As reviewed herein, cell type-targeting mouse models (include cre/lox technology), cell type-targeting viral manipulations (include RNA interference and possibly also CRISPR-based gene editing in the near future), single-cell RNA sequencing (allows to study gene expression profiling in diverse cell types and subpopulations), and analysis of protein expression in isolated cell type-specific samples (allows to study gene function and post-translational modifications critical for protein function) are increasingly used by glaucoma researchers to collect cell type-specific information. The molecular information is then validated and expanded by visualization and functional studies. Ongoing research pursues this integrating path for ultimate translation of the experimental data into new therapeutics for glaucoma patients.

### 2.2. Converging Etiological Paths to Evaluate for Unified Treatment Strategies

Even though current understanding of glaucomatous neurodegeneration is incomplete, it is undoubted that multiple pathogenic processes operate RGC degeneration, concurrently. The final phenotype of neurodegeneration in glaucoma includes the outcomes of biomechanical, vascular, metabolic, oxidative, and inflammatory stress. The multifactorial nature of glaucoma prohibits precision treatments approaching a single target. Instead, more significantly beneficial effects can be achieved with combination treatments targeting multiple etiological paths. Likewise, treatments with multitarget potential, which can simultaneously or successively manipulate multiple pathologies at multiple injury sites, can yield the best treatment efficacy to provide widespread structural protection and to preserve visual function in such a complex neurodegenerative disease. As outlined in this review, multitarget treatment strategies have a compelling pathophysiological basis for more efficient treatment of glaucomatous neurodegeneration. Among various neurodegenerative processes implicated in glaucoma, this review focuses on mitochondrial dysfunction and glia-driven neuroinflammation. This is because mitochondrial failure and inflammatory toxicity are major pathogenic components that may affect RGC axon, soma, and synapse health during glaucomatous neurodegeneration. Despite a vast diversity of insults, targeting these degenerative processes with shared impacts in the retina and optic nerve may offer a unified treatment strategy for protection of RGC axons, somas, and synapses.

It is also important to consider that there is an interdependence between mitochondrial and inflammatory paths of neurodegeneration. Dysfunctional mitochondria can stimulate glial inflammatory responses [44], aside from increasing the RGC susceptibility to inflammatory injury. On the other hand, proinflammatory mediators impair mitochondria and boost the neurodegenerative impacts of mitochondrial failure [45]. This relationship implies that therapeutic manipulation of mitochondrial function may also be immunomodulatory, while treatments targeting neuroinflammation may protect mitochondria against cytokine-mediated injury. Following sections outline recent experimental studies of mitochondrial dysfunction and glia-driven neuroinflammation with particular emphasis on the importance of these converging neurodegenerative paths for glaucoma treatment.

## 3. Neurodegenerative Consequences of Mitochondrial Dysfunction

Glaucoma-related stress, alongside aging, can damage mitochondrial structure and function, thereby reducing RGC survival through energy deficits, oxidative stress, and calcium imbalance. Mitochondria occupy a central place in the regulation of RGC apoptosis, axon degeneration, and inflammatory responses. IOP-related stress can disturb mitochondrial homeostasis by directly inducing calcium influx via mechanosensitive ion channels [12,14]. Biomechanical and vascular stress on axons at the optic nerve head may also cause early interruption of axoplasmic flow. Due to an essential role for axoplasmic flow in transferring of neuronal survival factors and trafficking of intracellular organelles, including mitochondria, axonal transport deficits may result in neurotropin deprivation, as well as advancing the mitochondrial failure. In turn, mitochondrial energy shortage may further compromise axonal transport that is a high energy-demanding cellular process [46,47,48]. Axonal disconnection triggers signaling for RGC soma death and distal axon degeneration. Survival factor insufficiency originated from an axonal insult at the optic nerve head is conventionally viewed as an early signal to provoke the apoptosis of RGC somas in glaucoma [49,50]. Apparently, decreased supply of survival factors can initiate a self-destruction program mediated by Bax [51,52]. Accordingly, several studies demonstrated beneficial outcomes of neurotrophins to protect RGCs in glaucoma models [53,54,55]. Ongoing clinical trials test the neuroprotective potential of neurotrophin support in glaucoma patients using either encapsulated cell implants to provide intravitreal sustained release of soluble ciliary neurotrophic factor, or eye drops for direct supplement of the nerve growth factor [54,56].

### 3.1. Mitochondrial Dysfunction and Metabolic Failure

Mitochondrial energy shortfall is recognized as an essential element of the neurodegenerative pathology in glaucoma [57,58]. Not only impeded trafficking of mitochondria [59], but glaucomatous RGCs also present dysregulation of various other processes that represent the framework of mitochondrial dynamics for adaptation to stress and increased energy requirements, which include mitochondrial biogenesis [46], morphology [60,61], and degradation [62]. Early intrinsic responses for synaptic rearrangements in the glaucomatous retina are also accompanied by mitochondrial redistribution in this highly energy-demanding region [26]. Consequently, a metabolic breakdown resulting from mitochondrial impairment is critical for RGC survival and visual function [57,58]. The high energy need of RGCs that possess long axons and extremely active metabolism for visual signaling, along with the high dependence of energy generation to mitochondria, make them particularly vulnerable to energy insufficiency caused by dysfunction of these cellular energy generators. Increased energy demand of stressed RGCs in glaucoma, accompanied by the aging-related reduction in energy reserves, may further augment the impacts of a metabolic failure. Besides mitochondrial shortage in ATP generation, elevated IOP-related biomechanical stress on capillaries, or IOP-independent vascular dysregulation, may also cause reduced supply of energy substrates from circulation, thereby collectively increasing the energy shortfall in RGCs [63,64,65].

Neuronal energy deficits in glaucoma may also be secondary to an insufficiency in glial metabolic support to RGCs. When the neuronal energy need increases, or glucose delivery and ATP generation decrease, astroglia and oligodendrocytes provide neurons with energy substrates through the glia-neuron energetic shuttle. By mobilizing their glycogen stores, these glial cells can supply neurons with glucose, or glycolysis-derived metabolic intermediates, such as lactate or pyruvate, via glucose or monocarboxylate transporters (MCT) [66,67,68,69]. Expression of lactate dehydrogenase that catalyzes the conversion between lactate and pyruvate supports the ability of RGCs to use these intermediates as bioenergetic substrates [70]. However, there appears to be an insufficiency or withdrawal of the glial metabolite delivery to RGCs in glaucomatous tissues. This is verified by a downregulated expression of MCTs in DBA/2J glaucoma, which was restored with a ketogenic diet that rescued the structure and function of RGCs and their axons [71]. In a more recent study, transgenic overexpression of MCT2 in RGCs also provided protection to these metabolically vulnerable neurons in DBA/2J glaucoma [72]. Since impaired energy metabolism is an important causative factor for RGC degeneration in glaucoma, an increasing number of studies investigate potential strategies to provide bioenergetic support to RGCs.

Short term hyperglycemia protected RGC somas and axons in a rat model with ocular hypertension [73], and subconjunctival injections of 50% glucose provided a temporary recovery of contrast sensitivity in a pilot clinical study [74]. In a following clinical trial, short-term topical treatment with the eye drops of concentrated glucose also improved contrast sensitivity in some patients with primary open-angle glaucoma [75]. This intriguing approach to depletion of metabolic resources in glaucoma raises exciting questions as to whether glaucomatous RGCs have the capacity to switch from respiratory to glycolytic metabolism. Oxidative phosphorylation and glycolysis can actually cooperate to maintain cellular energy balance [76]. Although cellular ATP generation in RGCs is primarily through mitochondrial oxidative phosphorylation, expression of both oxidative phosphorylation and glycolysis enzymes substantiates that these neurons have the machinery needed for both processes [58]. Relevantly, proteomics analysis of the ocular hypertensive human retinas displayed a prominent downregulation of many enzymes of oxidative phosphorylation, along with a parallel increase in the expression of glycolysis enzymes [77]. A hypoxic element evident by increased hypoxia-inducible factor-1 alpha (HIF1α) in the retina and optic nerve head of human donor eyes with glaucoma [78] and ocular hypertensive mouse eyes [79,80] may also provide a HIF1α-regulated mechanistic background for increased glycolysis. However, conflicting experimental observations [81] warrant this an area of further study to determine whether enhanced glycolysis in RGCs may compensate decreased ATP yields of mitochondria. It is also appealing to determine whether a metabolic shift between these two ATP-producing pathways, which may likely involve both RGCs and inflammatory glia in ocular hypertensive eyes, may help limit free radical generation from the respiratory chain, another important source for RGC stress in the glaucomatous retina and optic nerve [82].

A more recent study demonstrated dysregulated glucose metabolism and decreased levels of retinal pyruvate prior to detectable neurodegeneration in DBA/2J glaucoma. For testing pyruvate as a therapeutic energy source, this study examined the effects of oral pyruvate supplementation that resulted in a neuroprotective effect in both DBA/2J mice and a rat model of glaucoma [83]. An ongoing clinical study evaluates the synergistic effects of pyruvate and nicotinamide supplementation, detailed below, to provide protection for glaucoma.

### 3.2. Mitochondrial Dysfunction and Oxidative Stress

As being a major source for free radical generation, dysfunctional mitochondria cause oxidative stress when the production of reactive oxygen species (ROS) overcomes the intrinsic antioxidant response. By particularly considering the aging-related decrease in intrinsic antioxidant capacity, oxidative stress can deeply perturb RGC survival in glaucoma [48,82,84]. Amplified generation of superoxide in injured axons and then in RGC somas was suggested to serve as a signal for RGC apoptosis [85]. As highlighted below, this early insult initiated by mitochondrial dysfunction enhances cellular imbalances in nicotinamide adenine dinucleotide (NAD) and calcium, which are intimately linked elements of mitochondrial homeostasis and neuron survival. Moreover, neurodegenerative outcomes of oxidative stress in RGCs include oxidation of cellular macromolecules, modulation of protein function by redox modifications [86,87], and stimulation of neurodegenerative inflammation [88,89]. Many studies have recently targeted oxidative stress to improve RGC survival in animal models of glaucoma.

A natural antioxidant, α-lipoic acid, given to DBA/2J mice in their diet reduced the oxidative stress in ocular hypertensive retinas and increased RGC viability and axonal transport [90]. In another study, Tempol, a multifunctional antioxidant, was given through osmotic mini pumps for drug delivery by constant infusion in rats with experimental glaucoma. Antioxidant Tempol treatment protected RGC somas and axons against glaucomatous injury [91]. As agreed with the multiple roles of oxidative stress in neuroinflammation [88,89,92], findings of this study also detected immunomodulatory effects of antioxidant treatment in ocular hypertensive rat eyes [91]. Dietary supplement of coenzyme Q10, another antioxidant, also improved RGC survival and axon integrity and inhibited astrocyte reactivity in DBA/2J mice [93]. Likewise, coenzyme Q10 was found protective to RGCs in ocular hypertensive rat eyes after twice daily topical application for three weeks [94]. Many other studies that evaluated different antioxidant strategies in experimental or clinical studies detected mild to moderate protection against glaucoma, which were reviewed in detail elsewhere [95]. Clinical studies testing different nutrients or multivitamin complexes with antioxidant capacity are also reviewed in this Special Issue [96]. Experimental observations support the potential of antioxidants to provide neuroprotection and immunomodulation in glaucoma. However, additional and longer studies are needed for further testing.

The NAD, a redox cofactor that participates in many biochemical reactions critical for mitochondrial function, cellular metabolism, aging, and neurodegeneration [97,98], has recently been linked to glaucomatous neurodegeneration [79]. ATP-dependent conversion of nicotinamide mononucleotide to NAD declines with aging. This age-related depletion of cellular NAD bioavailability is exacerbated in DBA/2J mice, thereby rendering RGCs more susceptible to glaucomatous injury. Indeed, dietary supplement of NAD precursor nicotinamide (a form of vitamin B3) or overexpression of nicotinamide nucleotide adenylyltransferase 1 (NMNAT1) that is a key NAD-producing enzyme, prevented metabolic breakdown and rescued RGCs in mouse glaucoma [79,99]. Nicotinamide together with *Wld^S^* (that has a component of NMNAT1 overexpression [100]) robustly protected somatic, synaptic, and axonal compartments of RGCs in DBA/2J glaucoma [101]. This observation supports the NAD-boosting effect of *Wld^S^* in neuroprotection, in addition to protection against ATP depletion-related axon degeneration [102]. More recently, NAD decrease was also implicated in neuroinflammation-induced axon degeneration in glaucoma [103]. Pursuing these preclinical observations, a cross-over clinical study sought to assess the efficacy of oral vitamin B3 supplementation for 12 weeks. This study detected a trend towards improved function of the inner retina in glaucoma patients [104]. Further studies are underway to elucidate long-term treatment effects of nicotinamide and pyruvate supplementation on visual field defects.

In addition to insufficient production or increased cleavage, decreased bioavailability of NAD may also be related to over-consumption by NAD-consuming enzymes, including sirtuins. These NAD-dependent deacetylases play critical roles in DNA damage response [105] and provide beneficial effects against aging and age-related diseases. Consistently, treatment with resveratrol, a sirtuin activator, enhanced RGC survival in a mouse model with ocular hypertension [106].

### 3.3. Mitochondrial Dysfunction and Disturbed Calcium Homeostasis

In profound association with metabolic failure and oxidative stress, dysregulation of the cellular calcium homeostasis constitutes another neurodegenerative outcome of mitochondrial dysfunction in glaucoma. Impairment of NAD-dependent calcium regulation, aside from increased calcium influx through calcium channels or increased calcium release from intracellular stores, results in cellular calcium imbalance. Inefficient buffering of calcium causes calcium increase in RGCs as evident in a rat model of ocular hypertension [107] and activates calcium-dependent pathogenic processes. For example, dysregulated activity of the ubiquitin-proteasome system promotes protease-mediated cytoskeletal breakdown and destruction of the structural and functional integrity of axons [51]. Cellular calcium imbalance may also result in increased activities of calpains. Calpain-mediated proteolysis of axonal neurofilaments contributes to disintegration of axonal cytoskeleton during Wallerian degeneration [108], while boosted NAD production with *Wld^S^* [101] reduces axonal calcium and rescues axons. The calpains upregulated in glaucomatous tissues [109] may similarly contribute to RGC death in glaucoma [110].

An additional downstream consequence of calcium dysregulation is cellular degradation by autophagic activation. Autophagy is a physiological recycling process for adaptation to cellular stress and nutrient shortage. However, dysfunction of this cellular machinery may also lead to axon degeneration and RGC death in animal models of glaucoma [111,112,113,114]. In a recent study of DBA/2J mice, suppression of the mammalian target of rapamycin (mTOR), a cellular nutrient sensor, metabolic regulator, and suppressor of autophagy, diminished metabolic failure and neurodegeneration [83]. Activation of mitochondria-specific autophagy, called mitophagy, allows controlled turnover of damaged and dysfunctional mitochondria to eliminate mitochondria-originated neurodegenerative outcomes. Despite an increase in autophagic activity [115], DBA/2J glaucoma presented deficits in this mitochondrial quality control mechanism [62]. Dysfunction of mitophagy in ocular hypertensive rat eyes was partially eradicated by overexpression of E3 ubiquitin ligase parkin [116]. Additionally, deletion of mitochondrial uncoupling protein 2 in RGCs stimulated mitophagy and improved RGC survival in ocular hypertensive mice [117]. Another intrinsic mechanism that protects mitochondrial integrity is mitochondrial unfolded protein response that is regulated by mitochondria–nucleus communication to promote mitochondrial biogenesis and metabolic adaptation. This intrinsic response may also coordinate with mitophagy for mitochondrial recovery [118].

Of note, calcium dysregulation and redox imbalance are shared consequences of damaged mitochondria and endoplasmic reticulum stress. Specific stress response of endoplasmic reticulum, called unfolded protein response, aims for protein repair and targeted degradation of misfolded proteins to preserve cell survival and function. However, sustained stress of endoplasmic reticulum may also become a potential trigger for RGC degeneration in human glaucoma [77,109] and animal models [119,120,121,122].

Hence, mitochondrial dysfunction is a driving force behind multiple pathogenic processes of glaucomatous neurodegeneration. Treatments aiming to preserve and restore mitochondrial function and cellular metabolism certainly remain an active focus of glaucoma research to develop new therapeutics.

### 3.4. Mitochondria in RGC Degeneration

#### 3.4.1. Mitochondria in the Regulation of RGC Apoptosis

Multiple death pathways are coactivated in RGC somas, and their cross-talk may reinforce each other for RGC apoptosis in glaucoma. There is a consilience of evidence that these pathways converge to mitochondrial recruitment that is the point of no return in apoptosis. Mitochondrial apoptosis program is tightly regulated by a critical balance between the proapoptotic, antiapoptotic, and BH3-only members of the Bcl2 family [52]. Bax, the major proapoptotic member of the family, mediates the commitment step of RGC apoptosis. Once Bax is activated and translocated to mitochondria, following release of mitochondrial mediators initiates the proteolytic caspase cascade for execution of apoptosis. Bax deficiency provides complete protection to RGC somas and proximal axons in DBA/2J glaucoma [8,10]. Not only complete knock-out, but lowered quantity of Bax may protect against RGC apoptosis. Yet, Bax-depleted RGCs become quiescent and need rejuvenation to regain their functionality [10,123]. As discussed in the previous section, dysfunctional mitochondria caused by glaucoma-related biomechanical or vascular stress or neurotrophin deprivation promote Bax-dependent death of RGCs.

Besides the intrinsic pathway regulated by mitochondria, RGC apoptosis can also be mediated through the extrinsic pathway triggered upon ligation of tumor necrosis factor (TNF) family of dead receptors, such as TNFR1 [124,125] or Fas [126] by TNFα or FasL. The extrinsic pathway of RGC apoptosis through TNFR signaling may also recruit mitochondria, as caspase-8, initiator caspase of the receptor-mediated apoptosis, cleaves and activates Bid, a BH3-only protein [127]. This proximal caspase of the TNFR-mediated apoptosis pathway is activated in RGCs during neurodegeneration in human donor eyes [109] and animal models with glaucoma [127,128]. Likewise, cell type-specific analysis of experimental glaucoma detected cleaved caspase-8 in RGCs undergoing apoptosis [129]. Resulting from mitochondrial recruitment, TNFα-mediated RGC apoptosis involves proteolytic caspase cascade, mitochondrial dysfunction, and oxidative stress [86,109,124].

Caspases have been targeted to inhibit RGC apoptosis in different models of glaucoma-related RGC injury [86,124,130,131]. Although caspase inhibition provided some protection against apoptosis [86,124,130], rescued RGCs did not fully recover but eventually died due to mitochondrial impairment. This is because the cell death program reaches an irreversible point once Bax is activated in early stages [10,52], as underlined above.

Several upstream regulatory mechanisms may control the apoptotic function of Bcl2 family in RGCs [109]. For example, 14-3-3 proteins, checkpoint regulators of the cellular protein trafficking, control the subcellular localization and function of BH3-only proteins, such as Bad, in a phosphorylation-dependent manner. As evident in experimental glaucoma, the 14-3-3 scaffold may keep phosphorylated Bad sequestered in the cytoplasm, thereby preventing its mitochondrial translocation to neutralize antiapoptotic proteins [132].

#### 3.4.2. Mitochondria in the Regulation of Axon Degeneration

As previously mentioned, degeneration of RGC axons in glaucoma also involves the elements of mitochondrial dysfunction, including metabolic failure, oxidative stress, and calcium imbalance. Wallerian-type degeneration of distal axons in glaucoma [8,11,33,34] is an axonal self-destruction program characterized by cytoskeletal disassembly and granular degeneration, which is followed by the glial removal of axonal and myelin debris. It is evident that activation of sterile alpha and toll/interleukin-1 receptor (TIR) motif containing-1 (SARM1) induces metabolic failure and axon degeneration through Wallerian degeneration, and dying back, processes. SARM1 with NADase activity induces NAD loss and calcium influx and disturbs the energy metabolism in RGC axons, while its deletion rescues injured axons [133]. However, signaling for RGC soma death is independent from SARM1 [134]. Strikingly, TNFα-mediated neuroinflammatory signals can also stimulate SARM1/NAD-dependent axon degeneration, and subsequent oligodendrocyte loss and RGC death [103]. The loss of myelinating oligodendrocytes that maintain axonal integrity is critical for axon loss via trans-synaptic degeneration in glaucoma [135].

Although Bax is essential for RGC apoptosis but is not required for degeneration of axons, Bax deficiency slowed axon degeneration in DBA/2J glaucoma [10]. This observation may be explained by potential Bax involvement in intrinsic axon degeneration through death receptor-6 (DR6), another member of the TNFR superfamily [136]. Activation of DR6 by the surface ligands released after trophic deprivation [134] may induce axon degeneration that requires a downstream caspase cascade including caspase-6. However, this pathway linked to Alzheimer’s disease has not been explored in glaucoma models. Instead, a non-canonical form of necroptosis has recently been implicated in TNFα-induced SARM1/NAD-dependent axon neurodegeneration in glaucoma [103].

#### 3.4.3. MAPKs in Signaling for RGC Degeneration

Experimental studies to better understand how the initial axon injury signals for the apoptosis of RGC somas indicated Jun N-terminal kinases (JNKs), members of the mitogen-activated protein kinase (MAPK) family. Among different JNK isoforms that mediate physiological processes or stress signaling, JNK2 and JNK3 are major regulators of the RGC death after an axonal insult [137,138]. However, the JNK1-Jun axis integrates the upstream signals with a downstream transcriptional activity that controls RGC apoptosis after axon injury [138,139,140]. This is supported by the protection of RGC somas, but not RGC axons, against glaucomatous injury in Jun-deficient DBA/2J mice [140]. Importantly, Jun can be activated in the absence of JNK2 and JNK3 in these mice, thereby implicating JNK1 in RGC death [138].

**Figure 2:**
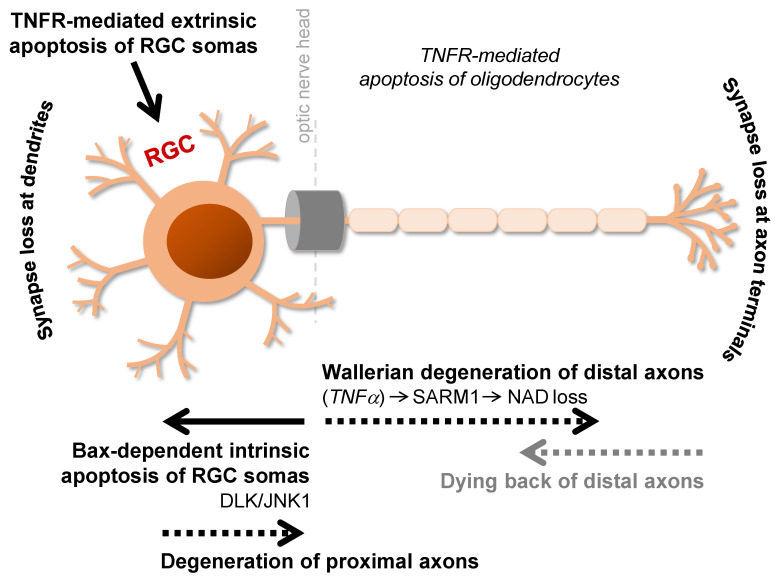
Distinct molecular programs regulate somatic and axonal degeneration of RGCs in glaucoma. Glaucomatous neurodegeneration involves RGC axons, somas, and synapses at dendrites and axon terminals. Optic nerve head is a critical site of injury, and early axonal insults may originate distal and proximal signals for axonal and somatic degeneration of RGCs. A distal axonopathy is processed through Wallerian degeneration and dying back, while degeneration of proximal axons is secondary to the apoptosis of RGC somas. The apoptotic death of RGCs is processed through intrinsic/mitochondrial and extrinsic/dead receptor-mediated pathways.

Jun may coregulate Bax-dependent RGC apoptosis with another proapoptotic transcription factor, DNA damage inducible transcript-3 (Ddit3) that is a key mediator of the endoplasmic reticulum stress response. Combined deficiency of Jun and Ddit3 reduced RGC apoptosis [141], but deletion of Ddit3 alone conferred only mild protection to RGC somas in DBA/2J glaucoma [122]. Distinct upstream kinases, such as MAP2Ks (including MKK4 and MKK7) and MAP3Ks (including DLK, a dual leucine zipper kinase) [142] regulate JNK activity in RGCs [142,143,144]. After axon injury, phosphorylated DLK can start a transcriptional program for RGC apoptosis by interacting with the Bcl2 family of genes [145]. In support of the DLK/JNK signaling as a critical regulator of RGC apoptosis, DLK deficiency abolished JNK activation in RGC somas and delayed RGC soma loss after axon injury but did not prevent axonal JNK activation or axon degeneration [142] (Figure 2). Additional studies of experimental glaucoma models are needed for further characterization of the JNK-mediated RGC death signaling; however, it is evident that various pathogenic processes implicated in glaucomatous neurodegeneration, including distortion of axonal cytoskeleton, neurotrophin insufficiency, mitochondrial dysfunction, metabolic failure, endoplasmic reticulum stress, or inflammatory signaling, can activate JNKs, as detected in human glaucoma and animal models [139,140,146,147,148].

Not only in the intrinsic apoptosis pathway triggered after axon injury, but JNK1 is also engaged in the extrinsic pathway of RGC apoptosis triggered after TNFR binding [147,149]. Intriguingly, inhibition of the initiator caspase-8 suppressed JNK1 signaling in RGCs, besides preventing RGC apoptosis in experimental glaucoma [150]. Several clues suggest that similar to RGC death induced by biomechanical or inflammatory stress, RGC death due to vascular stress is mediated through JNK signaling as well [151]. Interestingly, aside from TNFR or toll-like receptor (TLR) signaling, JNK1 can also prime inflammation via phosphorylation of the critical components of inflammasome [152] that is a multiprotein complex needed for the proteolytic activation and secretion of proinflammatory cytokines. These observations stimulate further research to fully assess the roles of different upstream regulators of JNK signaling in neurodegeneration and neuroinflammation to value them as treatment targets for glaucoma.

## 4. Glia-Driven Neuroinflammation in Glaucoma

### 4.1. Glial Responses to Glaucoma-Related Stress and Injury

Both astrocytes [129,153,154,155,156,157] and microglia [154,158,159,160,161,162] robustly respond to glaucoma-related stress by finely regulated morphological, molecular, and functional alterations. In addition to astrocytes and microglia, Müller glia in the retina [163], lamina cribrosa cells in the optic nerve head [164], and myelinating oligodendrocytes in the optic nerve [165,166] also prominently respond. The type and magnitude of tissue stress and neuron injury, spatial and temporal variations in glial responses, and intimate interactions of glial subtypes are critical determinants of the neurosupportive versus neurodegenerative outcomes of the RGC-glia relationship in glaucoma [36,37,38].

Glial cells that are critical regulators of neuronal homeostasis provide RGCs with structural, trophic, metabolic, and extracellular buffering support, modulate synaptic plasticity and transmission, regulate blood flow, maintain blood–brain barrier, and promote tissue healing by debris clearance via scavenger and phagocytosing functions. However, glial neurosupport functions may fail over extended periods of stress, and prolonged glial responses create an environment detrimental to RGCs. As well as innate neurotoxicity, expanded glial responses can stimulate adaptive immunity, through which T cells and autoantibodies reactive to ocular antigens may become damaging to RGCs during glaucomatous neurodegeneration [35,36,37,38]. Complement-mediated processes that are particularly involved in dendritic and synaptic degeneration of RGCs [167,168,169] may also contribute to the inflammatory phenotype [170]. Furthermore, besides resident glia in the retina and optic nerve, blood-born monocytes [171] are likely involved in early proinflammatory responses in glaucoma. This is supported by reduced neurodegeneration after inhibition of the monocyte extravasation by whole-body radiation [171], or pharmacological or genetic targeting strategies [172] in DBA/2J mice.

**Figure 3:**
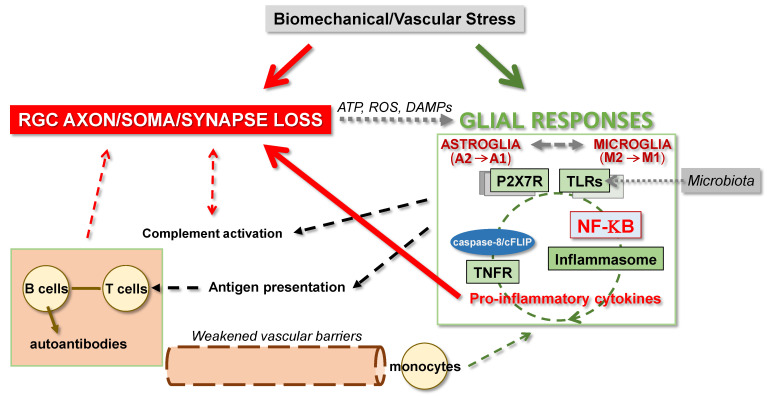
Glia-driven neuroinflammation can promote widespread injury to RGCs in glaucoma. Glial cells, including astroglia and microglia prominently respond to glaucoma-related tissue stress and injury. Glia can sense mechanical strain through mechanosensitive ion channels, and they can sense cellular stress by recognizing ATP, reactive oxygen species (ROS), and other damage-associated molecular patterns (DAMPs) released from RGCs. These signals stimulate inflammation through purinergic receptors (such as P2X7R), pattern-recognition receptors (such as TLRs), and inflammasome activation. Sustained release of proinflammatory neurotoxic cytokines, such as TNFα, contributes to RGC injury by activating dead receptor signaling that includes TNF receptors (TNFR). Evidently, multiple inflammation pathways activated in the glaucomatous glia are commonly regulated by the NF-κB-mediated transcriptional program. Glial reactivities also lead to complement-mediated and adaptive immune responses with neurodegenerative outcomes. A vicious cycle of these processes may intensify the inflammatory injury of RGCs at different subcellular regions.

Glial cells can directly sense IOP-related mechanical strain through mechanosensitive ion channels [173,174]. They can also sense cellular stress by recognizing the damage-associated molecular patterns (DAMPs) arising from RGCs and respond via purinergic receptor or TLR signaling, and inflammasome activation [175]. While ATP can intracellularly activate inflammasome assembly, extracellularly transported ATP via pannexin channels can also function as an intercellular transmitter to activate inflammasome through purinergic signaling [173,176,177]. As outlined in the next section, various other DAMPs originated from damaged mitochondria can similarly stimulate inflammatory signaling in glaucoma. Not only these intrinsic danger signals, but also pathogen-associated molecular patterns, relevant to microbiota, can activate glial TLRs [178,179] (Figure 3). Observations in animal models support early inflammatory responses that may be dependent or independent to RGC injury. For example, *Wld^S^* protects DBA/2J mice from axon degeneration; however, glial inflammatory responses continue [23]. In contrast, RGC soma protection in *Bax*^−/−^ mice is accompanied by attenuated responses of glial cells after optic nerve crush [180].

In addition to retina and optic nerve head, glial inflammatory responses may manifest throughout the visual pathway. As detected by MRI brain scanning and functional testing in glaucoma patients, glial responses are evident in posterior visual projections, where they mediate trans-synaptic damage [135]. Astrocyte and microglia responses may even be detectable in the contralateral eyes of glaucoma models with normal IOP [181,182,183] and may expand to upper projection sites of RGCs [184,185]. The bilateral spread of astrocytes may initially reflect their metabolic redistribution to support neurons [186]; however, this adaptive state may subsequently convert to neurodegenerative inflammation. Given that neuroinflammation can promote neuron injury in the entire visual pathway, immunomodulation offers a broadly beneficial treatment strategy to protect RGCs at different injury sites from the retina to the brain [36,37,38].

Therapeutic targeting of glia-mediated secondary injury processes may particularly be important in the clinical setting, since glaucoma is usually diagnosed at advanced stages when despite therapeutic control of IOP, neurodegeneration continues to progress through a vicious cycle of prolonged tissue stress, neuron injury, glial responses, sustained release of neurotoxic inflammatory mediators, and immune dysregulation. Recovery of mitochondrial health or supplement of bioenergetics may potentially be anti-inflammatory; however, many neurodegenerative outcomes of glial reactivity to biomechanical, vascular, or neuronal stress persist.

Among multiple glial subtypes, responses of astrocytes are rapidly produced, broadly manifest, and long-lasting. Widespread neurodegenerative impacts of astrocyte responses in glaucoma are not limited to inflammatory toxicity but also promote biomechanical, vascular, and excitotoxic injury [65,187]. These features, contrary to early but more transient and restricted responses of microglia, make the astrocytes excellent candidates for a potential treatment target to restore immune balance, enhance neuron survival, and improve the outcome of glaucoma. Yet, regardless of their individual roles, profound interactions of glial subtypes are remarkably important for neuroinflammation and neurodegeneration.

### 4.2. Glial Interactions for Neuroinflammation

Astrocytes and microglia play complementary roles in neuroinflammation. Along with their synchronized inflammatory responses to glaucoma-related stress and RGC injury, astrocytes and microglia can directly communicate and also relay the signals onto each other to regulate their phenotype and immune functions. For example, glaucoma-related alterations of astrocytes involve microglia-initiated conversion from a neurosupportive state (A2) to a proinflammatory and neurotoxic phenotype (A1) [188]. Similar to inflammatory responses in other neurodegenerative diseases or aging [189], microglia-derived IL1α, TNFα, and C1q can mediate the induction of proinflammatory and neurotoxic astrocytes in glaucoma. Indeed, triple knockout of these molecules (*IL1*^−/−^ *TNFα*^−/−^ *C1q*^−/−^) [190], or treatment with a glucagon-like peptide-1 receptor agonist, NLY01 [191], reduced A1 transformation and protected RGCs in ocular hypertensive mice. Although a global knockout model does not provide cell type-specific information or cannot preclude the contribution of blood-born elements, these observations support the neurodegenerative potential of glial inflammatory responses in glaucoma. Recent observations in experimental mouse glaucoma were also supportive of the bidirectional interactions of astrocytes and microglia. Resembling the microglial factors that can stimulate the phenotypic conversion of astrocytes, reactive astrocytes via nuclear factor-kappa B (NF-κB)-regulated cytokines can shape microglia responses as well [192]. As of note on the simplified categorization of glial phenotypes, astrocytes and microglia may, in fact, simultaneously present multiple reactive profiles that vary spatially and temporally. Therefore, their different polarization states should be considered on a dynamic spectrum, rather than two distinct populations.

### 4.3. Interplay between Mitochondrial Dysfunction and Neuroinflammation

Research advancements that have taken place over the past decade have opened up new avenues for better understanding of inflammation that presents an interdependence with mitochondrial dysfunction [44,45]. Mainly owing to the proteobacterial origin of mitochondria, the mitochondrial constituents released after increased membrane permeability can act as potent triggers of innate immune responses [44]. Besides ATP, mitochondrial DNA, particularly its oxidized form, exemplifies another DAMP originated from mitochondria [44]. Heat shock protein-60, a highly antigenic stress protein linked to autoimmunity in glaucoma [179,193,194], is also a mitochondrial chaperone. Among the mitochondria-originated stimulators of inflammation, oxidative stress has been well-explored in experimental models of glaucoma [89].

It appears that multiple routes to mitochondria-mediated inflammatory activation in glaucoma include increased generation of ROS [82,89]. Based on experimental observations in glaucoma models, oxidative stress stimulates glial cytokine production [88], while proinflammatory cytokines further damage mitochondria [86]. Oxidative stress end-products may also function as intrinsic ligands for glial TLRs, and binding of these pattern recognition receptors initiates inflammation signaling through a myeloid differentiation primary-response protein-88 (MyD88)-dependent pathway [92]. In addition, ROS play a costimulatory role in glial antigen presentation to T lymphocytes [88], and protein oxidation [87] can modify the antigenic features of retina and optic nerve proteins to stimulate autoimmunity [195]. Oxidative stress-dependent accumulation of advanced glycation end-products in glaucomatous tissues [196] may similarly act as an antigenic stimulus, as well as triggering the inflammatory RAGE signaling. Oxidative stress may also incite complement dysregulation in glaucoma [197]. Moreover, NF-κB that regulates glia-driven neuroinflammation [129,192] is a redox-sensitive transcription factor. Indeed, antioxidant treatment limited NF-κB activation and proinflammatory cytokine production in the ocular hypertensive rat retina and optic nerve [91]. Ketogenic diet that resolved energy compromise and oxidative stress and improved RGC survival in DBA/2J glaucoma [71] also ameliorated NF-κB-regulated inflammation [198]. Thus, owing to the broad impacts of oxidative stress in both direct injury to RGCs and secondary neurodegenerative outcomes of neuroinflammation, antioxidants may offer a multitarget strategy for glaucoma treatment, which warrant further investigation. Although multiple experimental or clinical studies tested short-term protective outcomes of different antioxidant strategies [95,96], immunomodulatory outcomes remain elusive.

**Figure 4:**
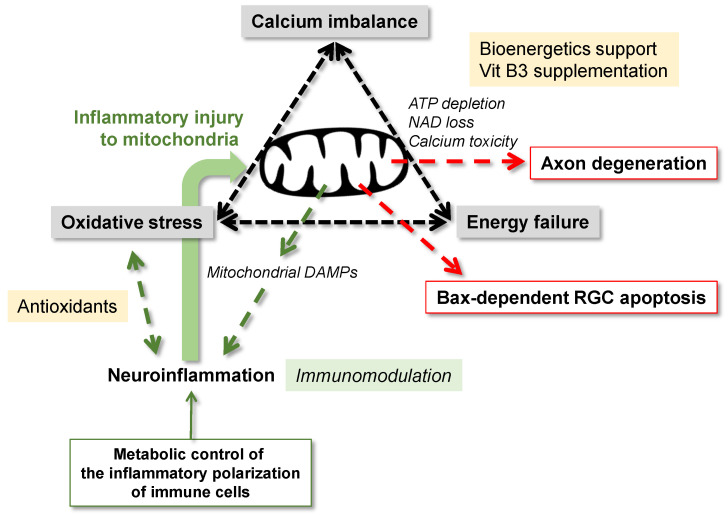
Interplay between mitochondrial dysfunction and neuroinflammation in glaucoma. Mitochondria through energy failure, oxidative stress, disturbed calcium homeostasis, and nicotinamide adenine dinucleotide (NAD) loss are critically involved in RGC degeneration and neuroinflammation. Mitochondria-generated oxidative stress and the mitochondrial constituents released after increased membrane permeability, including damage-associated molecular patterns (DAMPs), can induce glial inflammatory responses. Mitochondria’s role in neuroinflammation also includes the metabolic control of glial inflammatory polarization. While dysfunctional mitochondria induce neuroinflammation, proinflammatory cytokines may further impair mitochondria. Yellow boxes indicate the related treatment strategies that are being tested in clinical studies. As shown in the green box, immunomodulatory treatments are still explored in preclinical studies.

Mitochondria, by controlling the cellular metabolism, also play critical roles for inflammatory polarization of immune cells. Evidently, inflammatory stimuli switch a metabolic program resulting in increased glucose uptake and glycolysis and decreased oxidative phosphorylation, while mitochondrial oxidative metabolism is linked to an anti-inflammatory program [199,200] (Figure 4). These suggest that the modulation of bioenergetic pathways may resolve inflammation. This possibility is also motivating to further study. Although preclinical studies investigating the bioenergetic support provide an encouraging trend for neuroprotection in glaucoma [71,79,83], treatment results on inflammatory responses should also be evaluated.

### 4.4. Molecular Players of Neuroinflammation in Glaucoma

Since glia-produced proinflammatory mediators are neurotoxic and exacerbate RGC degeneration in glaucoma, therapeutic modulation of neuroinflammation can protect RGCs from inflammatory toxicity [36,37,38]. There is no doubt that enhanced molecular understanding of immune regulation is the key to open the way to a therapeutic approach by immunomodulation. Towards this goal, recent transcriptomic or proteomic profiling of the inflammatory responses in animal models or human donor tissues with glaucoma have revealed an early upregulation of numerous molecules. These molecules consisted of sensors/inducers (such as purinergic receptors, TLRs, or inflammasome sensors, nucleotide oligomerization domain-like receptors), transducers/regulators (such as MAPKs, NF-κB, or a TLR adaptor, MyD88), and amplifiers/effectors (such as cytokines and chemokines) of inflammation signaling [38,109].

A proinflammatory cytokine imbalance in glaucoma is best exemplified by increased glial production of TNFα that functions as the amplifier of neurodegeneration and effector of RGC injury [125,201,202]. Evidently, this proapoptotic cytokine secreted from reactive glia induces RGC apoptosis, oligodendrocyte death, and axon degeneration through TNFR1 signaling in in vitro [124] and in vivo models [103,147,203,204]. Proinflammatory activities of TNFα/TNFR1 signaling is considerably stronger, as downstream NF-κB activation and transcription of other immune mediators may feed into a vicious cycle [88,109,129,192]. Not only TNFα [124,147], but also FasL, secreted by reactive glia, may induce RGC loss by binding Fas receptor from the same family of receptors [126].

Several studies have tested different molecules as immunomodulatory treatment targets for glaucoma. For example, blocking the proinflammatory cytokine signaling using inhibitors of TNFα [204] or Fas receptor [126] reduced RGC death and axon loss in ocular hypertensive animals. In addition, deactivation of microglia by minocycline [205,206,207] delayed neurodegeneration in experimental glaucoma. More recently, NLY01 treatment inhibited proinflammatory activity of astrocytes and protected RGCs against glaucomatous injury in mice [191]. Because of the potential off-target effects of these treatments on systemic immune defense and glial neurosupport, alternative strategies for more specific targeting are still in exploration.

#### 4.4.1. Transcriptional Regulation of Glia-Driven Neuroinflammation

On the basis of astroglia-specific analysis, NF-κB, the main transcriptional activator of inflammatory mediators was among the pathways most affected in glaucoma [109,129]. Obviously, multiple inflammation pathways activated in the glaucomatous glia, including cytokine signaling, TLR signaling [92,129,162], and inflammasome assembly [109,129,177,208], are commonly regulated by the NF-κB-mediated transcriptional program [129,192]. Major proinflammatory cytokines secreted by glial cells in glaucoma, including TNFα or FasL are transcriptional targets for NF-κB. Many other proinflammatory cytokines, or chemokines that exhibit glial upregulation in glaucoma are NF-κB’s target genes as well. These include IFNγ, IL1, IL2, IL12, IL17 [175,184,209,210], and iNOS that can also cause collateral damage [211]. Additionally, NF-κB-regulated immunoreceptors, such as MHC molecules [212] or RAGE [196] that are involved in antigen presentation for systemic immunity, are among the glial molecules upregulated in glaucoma [35,38]. Even more, tenascin that is an extracellular matrix molecule upregulated in the glaucomatous optic nerve [213] is regulated by NF-κB and serves as an intrinsic ligand to TLRs [214]. It is notable that besides proinflammatory mediators, several other gene targets of NF-κB, which take part in glial extracellular matrix remodeling and profibrotic processes, may also participate in glaucoma-related biomechanical and vascular stress at the optic nerve head [2].

As being a common regulator of multiple inflammation and degeneration pathways, NF-κB appears to be a favorable target for immunomodulation and neuroprotection in glaucoma. A recent study therefore focused on astroglial NF-κB to value its potential as a treatment target. This study analyzed neuroinflammatory and neurodegenerative outcomes of ocular hypertension in mice with or without cre/lox-based conditional deletion of IκKβ in glial fibrillary acidic protein (GFAP)-expressing astroglia (GFAP/*IκKβ*). Deletion of astroglial IκKβ, the main activating kinase involved in IκB degradation via the canonical pathway of NF-κB activation, led to immunomodulatory outcomes [192]. The proinflammatory cytokines known to be transcriptional targets for NF-κB (including TNFα, IFNγ, IL1, and IL2) exhibited decreased production in the retina and optic nerve of ocular hypertensive GFAP/*IκKβ* mice. This study also revealed non-cell-autonomous functions of NF-κB in astroglia-microglia interactions in experimental glaucoma, as IκKβ deletion in astroglia reduced proinflammatory cytokine production in microglia as well [192]. This observation stimulates further studies to better characterize NF-κB-regulated processes in individual contributions and the cross-talk between astroglia and microglia during neuroinflammation. As a result of its immunomodulatory outcomes, inhibition of astroglial NF-κB provided structural and functional protection to RGCs [192]. Previous studies of other neurodegeneration models have also implicated NF-κB-mediated inflammation in neuronal injury [215,216] and synaptic degeneration [217]. Yet, given the pleitropic functions of NF-κB signaling, long-term effects of NF-κB inhibition on astrocyte plasticity and neurosupport functions should be further evaluated.

Although preclinical studies support the contribution of inflammatory processes as an important component of neurodegeneration in glaucoma and point to NF-κB the main transcriptional activator of glia-driven neuroinflammation and secondary injury processes, clinical modulation of NF-κB may be complicated. This is because NF-κB also activates critical antiapoptotic genes [218,219] and regulates a wide range of processes crucial for neuron survival, synapse formation, and plasticity [220,221], or systemic immunity. A hundred of reported inhibitors of NF-κB lacking the cell type specificity interferes with its physiological activities in neuron survival. Therefore, glia-targeting manipulation of NF-κB is favored to avoid off-target effects while inhibiting neurodegenerative inflammation in glaucoma. A treatment approach specifically targeting glia can eliminate the risk for undesired side effects on neurons, while keeping the systemic immune defense intact. Continuously emerging gene engineering techniques and new delivery tools, including astroglia-specific viral vectors [222,223,224,225,226,227,228], rise research expectations to enable clinical translation of the experimental information into glia-targeting strategies to prevent or reverse neurotoxic inflammation for glaucoma treatment. Nevertheless, with respect to spatial and temporal diversity of glial reactivity and functional consequences, glia-targeting treatments should carefully consider many different aspects.

#### 4.4.2. Other Regulators of Neuroinflammation

Based on diverse biological functions of caspase-8 [229], a recent study also searched for caspase-8 as a treatment target to ameliorate NF-κB-regulated inflammation signaling in experimental glaucoma. While enzymatic activity of caspase-8 upon TNFR binding triggers apoptosis as detected in the glaucomatous RGCs [109,127,128], its catalytic activity can promote NF-κB-mediated cell survival and inflammation [230,231] as detected in the glaucomatous glia [109,129,192]. Similar to TNFRs, including TNFR1 and Fas, ligation of TLRs can activate caspase-8-mediated inflammation [232,233,234,235]. Inflammasome activation also involves caspase-8-mediated processes [236,237,238,239]. Thus, besides RGC apoptosis, caspase-8 is engaged in various inflammation pathways linked to glaucoma as well.

In order to dissect cleavage-dependent and cleavage-independent functions of caspase-8 in different cell fates, namely RGC apoptosis and astroglia-driven neuroinflammation, two experimental approaches were used in parallel [150]. In the first approach, pharmacologic inhibition of caspase-8 cleavage in ocular hypertensive rat eyes protected against RGC apoptosis but did not affect the neuroinflammatory potential of glia. However, cre/lox-based conditional deletion of astroglial caspase-8 in the second approach reduced neuroinflammation and its neurodegenerative outcomes but induced astroglial necroptosis (due to complete lack of caspase-8 that is needed to prevent necroptotic activity). Cell type-specific diverse functions of caspase-8 in RGC apoptosis and astroglia-driven neuroinflammation may support its multitarget potential for glaucoma treatment. Findings of this study also indicated that the distinct functions of caspase-8 are regulated by FLICE-like inhibitory protein (cFLIP), another gene target for NF-κB [150]. This inactive caspase-8 homolog makes a cytosolic complex with caspase-8 after TNFR or TLR binding and acts as a molecular switch between cell death, survival, and inflammation signals [230,240,241,242,243]. Contrasting RGCs, cFLIP is highly expressed in astroglia and inhibits caspase-8-mediated cell death but induces proinflammatory outcomes [235,244]. The caspase-8/cFLIP interaction may be an alternative treatment target to modulate NF-κB-regulated inflammation signaling, while eliminating any undesired effects on NF-κB-regulated protective processes. This relationship awaits further studies.

Taken together, therapeutic modulation of neuroinflammation by targeting astroglial NF-κB [192], or caspase-8 [150], resulted in both structural and functional protection of RGCs in experimental glaucoma. Consistently, RGCs spared in ocular hypertensive mice by triple knockout of IL1α, TNFα, and C1q were electrophysiologically functional [190]. These observations suggest that immunomodulation may offer functional recovery to RGCs, unlike inhibition of RGC apoptosis [8,10,123]. This aspect is highly encouraging to further seek immunomodulatory strategies for glaucoma treatment.

## 5. Conclusions

As outlined herein, progressive degeneration of RGCs in glaucoma involves multiple triggers, multiple injury sites, multiple cell types, and multiple signaling pathways over a chronic disease period. Various etiological paths for glaucomatous neurodegeneration, including biomechanical, vascular, metabolic, oxidative, and inflammatory components, are continuously explored towards new treatment possibilities for this blinding disease. Growing information about intrinsic responses of RGCs, and the individual contributions and intimate interactions of glial subtypes provides a broader perspective into complex processes of neurodegeneration in glaucoma. Apparently, multitarget treatment strategies can allow to more efficiently manipulate multiple pathogenic processes for widespread protection in such a complex neurodegenerative disease. In order to achieve this goal, glaucoma research will pursue experimental studies to inhibit prodeath signals, stimulate prosurvival signals, and modulate neuroinflammation using pharmacological drugs, gene-based treatments, or stem cell-based approaches. Neuroregenerative therapeutics also seem promising to reprogram rescued RGCs for functional recovery. With the advent of powerful research tools, preclinical studies will more comprehensively identify and functionally validate new treatment targets for clinical testing. Undoubtedly, collaborative efforts of basic and clinician scientists along a path from bench-to-bedside and back will enable prompt translation of the accumulated information for ultimate success in preventing blindness from glaucoma.

## Data Availability

Not applicable.
